# Duodenal Diverticular Perforation after Small Bowel Obstruction: A Case Report

**DOI:** 10.1155/2018/6197828

**Published:** 2018-08-07

**Authors:** Khuram Khan, Saqib Saeed, Haytham Maria, Mohammed Sbeih, Farhana Iqbal, Alexius Ramcharan, Brian Donaldson

**Affiliations:** ^1^Department of Surgery, Harlem Hospital Center, Columbia University, New York, NY, USA; ^2^Department of Medicine, Richmond University Medical Center, Staten Island, New York, NY, USA

## Abstract

**Introduction:**

Duodenal diverticulum is a rare disease that can be easily missed. The incidence of duodenal diverticulum diagnosed by upper GI study is approximately 5%. Autopsy results show that 22% of the population have duodenum diverticulum. Most patients with duodenal diverticulum are asymptomatic. However, complications like inflammation, perforation with retroperitoneal abscess, sepsis, pancreatitis, bile duct obstruction, and bleeding can occur. Approximately 162 cases of perforated duodenal diverticulum have been reported in the literature.

**Case Presentation:**

We present a rare case of an 82-year-old female with perforation of a duodenal diverticulum caused by small bowel obstruction; in addition to this, there was a synchronous colonic tumor.

**Conclusion:**

Diagnosis and management of this rare disorder are controversial. Nonoperative management is advocated in some cases. Some of the cases require early aggressive surgical intervention. The mortality rate remains approximately 45% in all these patients.

## 1. Introduction

Perforation of the duodenum diverticulum (DD) is a rare disease that can be easily missed as it can be a clinical diagnosis. Management of the perforation requires urgent surgical intervention for favorable outcomes. Perforation can be caused by infections such as diverticulitis, ulceration, foreign body, trauma, and iatrogenic perforation after ERCP and would require CT scan of the abdomen for diagnosis and treatment in some cases, while diagnosis of duodenal diverticulum can be made with upper gastrointestinal series.

## 2. Case Presentation

**Figure 1:**
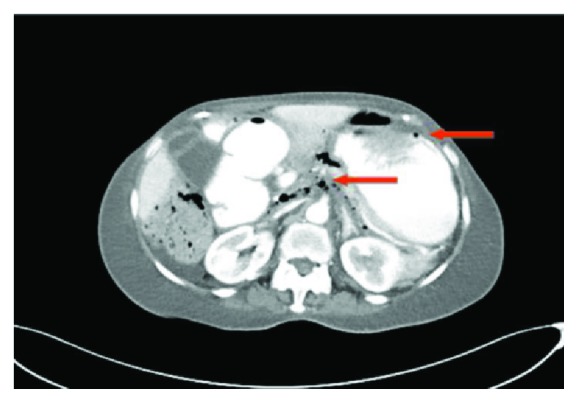
CT scan of the abdomen. Retroperitoneal free air.

**Figure 2:**
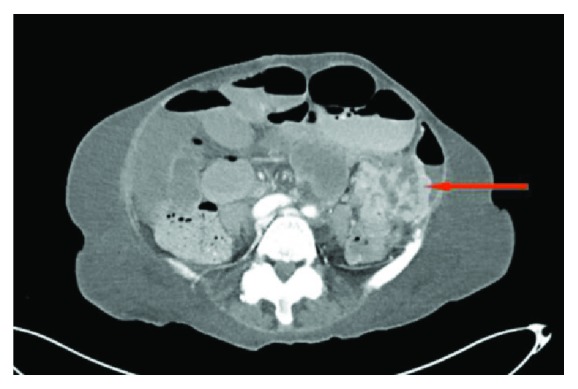
CT scan of the abdomen. Image shows dilated small bowel loops with air fluid level and small bowel obstruction.

**Figure 3:**
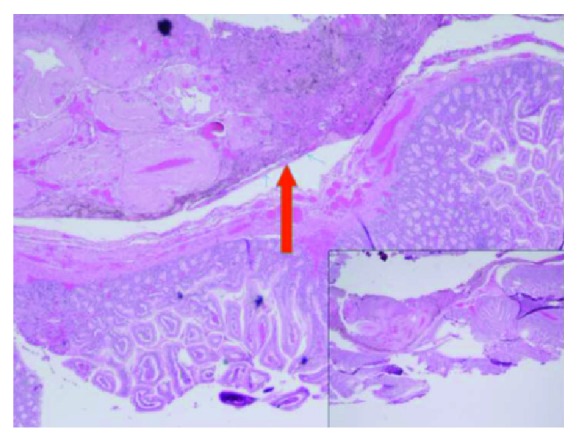
Histology slide. Small intestinal tissue with acute serositis (red arrow) consistent with perforated viscus.

An 82-year-old African American female with history of hypertension, chronic active smoker for 60 years along with prior surgical history significant for a laparotomy more than 20 years previously for unknown reason who was initially admitted to the medical service after a fall. She had a long history of nonspecific lower abdominal pain. As per her family, she had not seen a doctor for 10 years and never had a colonoscopy. She reported unintentional weight loss. Vital signs at the time of presentation were stable. On physical examination, she appeared cachectic and dehydrated. Her abdomen was soft, non-tender with audible bowel sounds. Mild right lower quadrant tenderness was noted. Labs were significant for microcytic hypochromic anemia and urine analysis positive for leukocyte esterase. Liver function test was normal. Chest X-ray showed cardiomegaly. Abdominal US revealed mild ascites and dilated common bile duct to 1 cm. The patient was admitted to medical service with a diagnosis of dehydration, failure to thrive, and for work-up of an occult gastrointestinal malignancy. She was scheduled for EGD and colonoscopy by gastroenterology team. In addition to all of this, her CEA was 12.2 ng/ml (normal less than 3 ng/ml). While the patient was on the medical service, her hemoglobin dropped to 6.2 gm/dL requiring blood transfusions. During the second unit of blood transfusion, the patient became hypoxemic and tachypneic. She was transferred to Medical Intensive Care Unit (MICU) and subsequently intubated for acute respiratory failure. Chest X-ray at this point showed bilateral infiltrates, and the patient was started on IV antibiotics for possible pneumonia. The scheduled GI procedures were cancelled due to critical health status. She had echocardiography in MICU which revealed mitral stenosis and severe pulmonary hypertension, with normal ejection fraction. Her respiratory status improved, and she was transferred back to medical floor after staying four days in MICU. She also had urine culture which grew klebsiella. Three days later after being transferred from MICU, she developed abdominal distension. A CT scan of the abdomen without contrast was obtained which revealed gastric, small bowel, and colonic distension. There was copious amount of stool in the colon. She was transferred to the surgical service for management of possible ileus/stool impaction pending colonoscopy to rule out colonic lesion. She was managed with nil per os, nasogastric tube suction, intravenous fluid hydration, and enemas. Nasogastric tube output was minimal but the patient's abdomen remained distended while on surgical service. The patient became hypotensive and tachycardic and less responsive. After fluid resuscitation, another CT of the chest and abdomen with oral and intravenous contrast was performed one week from prior CT, which revealed persistent dilation of the stomach, small bowel, and large bowel, a left colonic mass, and large amount of retroperitoneal free fluid in the region of the duodenum and pancreas (Figures 1 and 2). She was taken to the operating room for exploratory laparotomy. A midline laparotomy was performed, and numerous adhesions from the previous laparotomy were seen. The patient had a high-grade small bowel obstruction with a transition point in the mid ileum caused by adhesions. Lysis of adhesions was performed. There was a large left descending colonic mass invading the lateral abdominal wall causing partial large bowel obstruction. The transverse colon was attached to the left colon mass with adhesions, creating a space through which there was herniation of dilated small bowel. However, this did not cause an obstruction. These dilated bowel loops were reduced. Because of the finding of free air in the retroperitoneum of the upper abdomen, we decided to explore that area. Generous kocherization of the duodenum was performed. The head of the pancreas and hepatic flexure were mobilized and found to be normal. With further mobilization of the duodenum, a perforated duodenal diverticulum was noticed in the third part of the duodenum. There was a leak of bile in the area. There was extensive retroperitoneal necrosis extending across the upper abdomen to the tail of the pancreas. The duodenal diverticulum was broad based, inflamed, and closely attached to the superior mesenteric artery. The diverticulum was dissected off, stapled, and sent for pathology (Figure 3). The third part of the duodenum became dusky, and we decided to resect it with a GIA. At this point, the patient was hypotensive and acidotic. A decision was made to perform damage control and a temporary abdominal closure and to transfer the patient to ICU for resuscitation. In the ICU, she was aggressively resuscitated with correction of her acidosis, coagulopathy, and hypothermia. She was brought back to the OR the following day. There were no signs of bowel ischemia, and the small bowel obstruction had resolved. An end to side duodenojejunostomy was performed using an EEA. The anastomosis was protected with a Stamm's gastrostomy through which a feeding jejunostomy was passed distal to the anastomosis. A drain was left in the right upper quadrant. We then performed a transverse colon loop colostomy to divert the colonic tumor. Blood cultures later grew pseudomonas and yeast. The patient subsequently developed multiorgan failure, and the family requested DNI/DNR. Their after family requested terminal extubation 12 days after the last operation.

## 3. Discussion

Duodenal diverticulum complications are rarely seen, and it is the second most common site of diverticula of the gastrointestinal tract after the colon [1]. DD can be congenital or acquired [2]. The acquired type is usually a pseudodiverticulum where there is herniation of the mucosa through the wall of the duodenum. Most of these occur along the mesenteric border of the duodenum [3]. Duodenal diverticulum is not rare, but complications like perforation, bleeding, pancreatitis, and bile duct obstruction are very rare. Less than 200 cases of perforated duodenal diverticulitis have been reported in the literature [2]. Causes of perforation include diverticulitis, ulceration, foreign body, trauma, and iatrogenic perforation after ERCP. In our case, we believe that the perforation occurred secondary to duodenal distension as a result of back pressure from associated bowel obstruction. The diagnosis is difficult as patients present with nonspecific signs and symptoms. Usually, there is no tenderness on abdominal exam because of retroperitoneal perforation. Computerized tomography plays an important role in diagnosis by showing free retroperitoneal air and phlegmon. Upper GI studies and endoscopy can be used in stable patients. The diagnosis is usually made with intraoperative exploration, as in our case. Because of rarity of the disease, there are no clear guidelines regarding conservative versus surgical management. Some cases of known perforated duodenal diverticulum in stable patients were managed conservatively by nil per os, intravenous fluid hydration, intravenous antibiotics, parenteral nutrition, and percutaneous drains for the localized collections.

Many operative techniques have been described. After adequate mobilization of the duodenum, the diverticulum should be dissected completely down to the base and either handsewn or stapled diverticulectomy can be performed. The defect can be closed in layers [3]. The relation of the neck of the diverticulum to the common bile duct must be ascertained to avoid bile duct injury. The bile duct should be cannulated from either the ampulla of Vater, by making a choledochotomy, or through the cystic duct [4]. Simple closure of the perforation with drainage, without diverticulum resection, because of close proximity of the bile duct to the diverticulum, was described in one case report [5].

If the perforated duodenal diverticulum is in the fourth portion of the duodenum, partial duodenectomy can be performed and reconstruction with end-to-side doudenojejunostomy [2].

In the presence of significant inflammation, more complex procedures have been described. A Roux-en-Y loop of the jejunum is brought through a rent in the transverse mesocolon, the duodenum is transected, and the duodenal stump is oversewn. The Roux limb of the jejunum was anastomosed end to end to the duodenum. A jejunojejunostomy is created 40 cm distal to the duodenojejunostomy. A drain should be placed. As an alternative, vagotomy, antrectomy closure of duodenal stump, and Billroth 2 reconstruction can be performed. This decreases the risk of duodenal fistula [3]. For large duodenal diverticulum causing dilation and obstruction of the bile duct, choledochoduodenostomy is the best approach and avoids the complications associated with attempts to resect the diverticulum. Sphincteroplasty has been described as well [4]. For duodenal diverticulum causing bleeding, preoperative localization with endoscopy or angiography should be done. Excision of the diverticulum with suture ligation of the bleeding point is essential [6].

## 4. Conclusion

Duodenal diverticular perforation can be challenging as it is a rare disease and difficult to diagnose. Nonoperative management is only in selected patient's population. CT scan can help diagnose the perforation and require urgent surgical intervention.
